# Correlation of *MET* gene amplification and *TP53* mutation with PD-L1 expression in non-small cell lung cancer

**DOI:** 10.18632/oncotarget.24455

**Published:** 2018-02-08

**Authors:** Maher Albitar, Sucha Sudarsanam, Wanlong Ma, Shiping Jiang, Wayne Chen, Vincent Funari, Forrest Blocker, Sally Agersborg

**Affiliations:** ^1^ NeoGenomics Laboratories, Aliso Viejo, CA, USA

**Keywords:** NSCLC, PD-L1, EGFR, MET, TP53

## Abstract

**Background:**

The role of *MET* amplification in lung cancer, particularly in relation to checkpoint inhibition and *EGFR* WT, has not been fully explored. In this study, we correlated PD-L1 expression with *MET* amplification and *EGFR*, *KRAS*, or *TP53* mutation in primary lung cancer.

**Methods:**

In this retrospective study, tissue collected from 471 various tumors, including 397 lung cancers, was tested for *MET* amplification by FISH with a *MET*/centromere probe. PD-L1 expression was evaluated using clone SP142 and standard immunohistochemistry, and *TP53*, *KRAS*, and *EGFR* mutations were tested using next generation sequencing.

**Results:**

Our results revealed that PD-L1 expression in non-small cell lung cancer is inversely correlated with *EGFR* mutation (P=0.0003), and positively correlated with *TP53* mutation (P=0.0001) and *MET* amplification (P=0.004). Patients with *TP53* mutations had significantly higher *MET* amplification (P=0.007), and were more likely (P=0.0002) to be *EGFR* wild type. There was no correlation between *KRAS* mutation and overall PD-L1 expression, but significant positive correlation between PD-L1 expression and *KRAS* with *TP53* co-mutation (P=0.0002). A cut-off for the ratio of *MET*: centromere signal was determined as 1.5%, and 4% of lung cancer patients were identified as *MET* amplified.

**Conclusions:**

This data suggests that in lung cancer both *MET* and *TP53* play direct roles in regulating PD-L1 opposing *EGFR*. Moreover, *KRAS* and *TP53* co-mutation may cooperate to drive PD-L1 expression in lung cancer. Adding MET or TP53 inhibitors to checkpoint inhibitors may be an attractive combination therapy in patients with lung cancer and MET amplification.

## INTRODUCTION

In 2017, there will be approximately 220,000 new cases of lung cancer in the United States with approximately 160,000 deaths [1]. Platinum-based chemotherapy delivers a response rate of 20–35% and a median overall survival (OS) of 8–12 months in non-small cell lung cancer (NSCLC) [2, 3]. Improved understanding of the molecular signatures of NSCLC has led to the development of targeted therapeutics against epidermal growth factor receptor (*EGFR*), Kirsten rat sarcoma viral oncogene homolog (*KRAS)*, or anaplastic lymphoma kinase (*ALK)* alterations which can result in significant response and extended OS. However, a significant percentage of NSCLC patients do not have a targetable mutation. Moreover, the 5 year OS for NSCLC, even with targeted therapeutics, is 15% [4].

The blockade of immune checkpoints, through inhibition, such as programmed cell death (PD-1)/programmed cell death ligand (PD-L1) targeting, has shown remarkable response in some patients with NSCLC [5]. Pembrolizumab, a PD-1 inhibitor, is approved for first and second line PD-L1 expressing NSCLC, after demonstrating its association with significantly improved OS compared to docetaxel [6]. Nivolumab, another PD-1 inhibitor, and atezolizumab, a PD-L1 inhibitor, are approved to treat *ALK*/*EGFR* WT and mutant metastatic NSCLC [7, 8]. Higher PD-L1 expression is associated with better efficacy of PD-1/PD-L1 therapeutics and improved response in NSCLC, [9, 10] however PD-L1 expression is not the sole determinant of response to PD-1/PD-L1 therapy and some patients who do not express PD-L1 have been shown to respond to PD-1/PD-L1 inhibition [11, 12]. While PD-1/PD-L1 inhibitors can be remarkably effective in some patients producing durable responses, only a subset of patients will benefit (e.g. 10-40% of NSCLC) [7, 13].

Attempts have been made to establish a correlation between PD-1/PD-L1 expression and NSCLC driver dysregulation [4, 14, 15]. About 50–65% of patients with *EGFR*-mutated NSCLC respond to EGFR tyrosine kinase inhibitors (TKIs), [16] but most patients become resistant to these treatments by acquiring a secondary point mutation in *EGFR* or activation of additional signaling pathways, including mesenchymal-epithelial transition factor (*MET*) and *KRAS* [17, 18]. Dysregulation of MET signaling has been shown to drive tumorigenesis in NSCLC [19]. Inhibition of MET signaling in EGFR TKI-resistant cells may restore sensitivity to EGFR inhibitors [20, 21]. Additionally, anti-tumor activity of agents targeting MET has been observed in *MET*-amplified *EGFR* WT patients [22]. The correlation between *MET* amplification and PD-L1 expression, particularly for *EGFR* WT NSCLC patients is important for therapy and has not been fully explored.

To improve on the success of checkpoint inhibitors as monotherapy, combination therapy is being considered. Conventional chemotherapy and targeted therapy are currently being considered in combination with checkpoint inhibitors [23]. Theoretically, direct correlation between two molecular abnormalities suggests collaboration and selection between these two abnormalities and targeting both drivers may be more effective as a therapeutic approach. Toward this end, we explored the correlation between PD-L1 expression and *KRAS*, *EGFR*, *TP53*, and *MET* alterations in this study.

MET is a receptor protein-tyrosine kinase for hepatocyte growth factor, which induces the *RAS-ERK/MAPKs* cascades through the activation of Grb2-associated binding protein 1 (Gab1) [24]. Similarly, the EGFR is a receptor tyrosine kinase that induces *RAS-ERK/MAPK* and *AKT-PI3K-mTOR* [25]. Furthermore, inactivation of the *TP53* gene is well documented to cooperate with activated *RAS* to induce tumorigenesis [26].

## RESULTS

### PD-L1 expression

**Figure 1:**
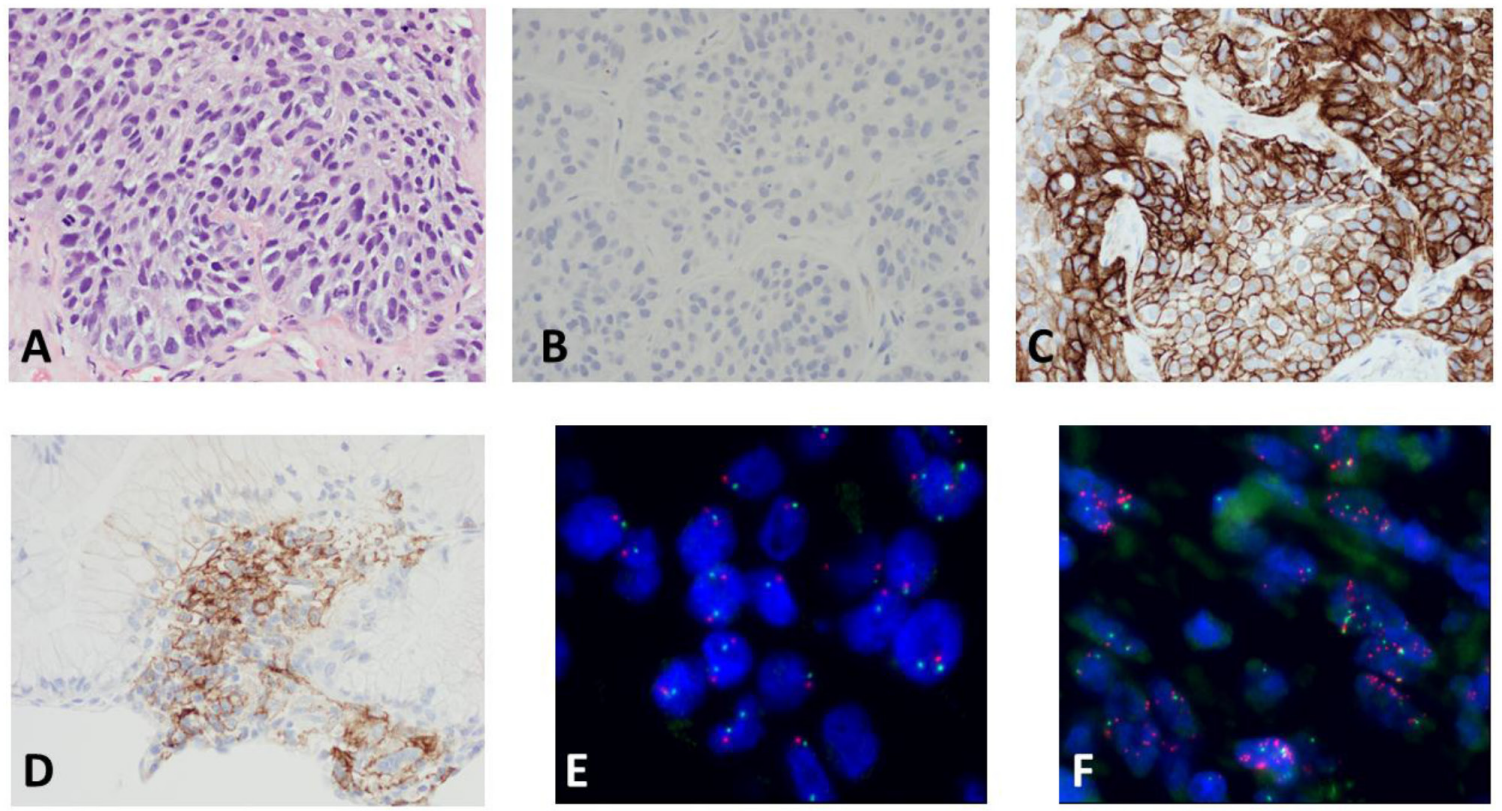
Representative examples of PD-L1 protein expression by IHC and MET amplification by FISH Top panel shows hematoxylin-eosin stain of the tumor in **(A)**, negative isotopic control in **(B)**, and PD-L1 staining in **(C)**. Intermediate PD-L1 staining in a different tumor is shown in **(D)**. The FISH images show MET amplification (red probe) in one tumor in **(E)** and no amplification in a different tumor in **(F)** The green signal represents chromosome 7 centromere.

**Figure 2:**
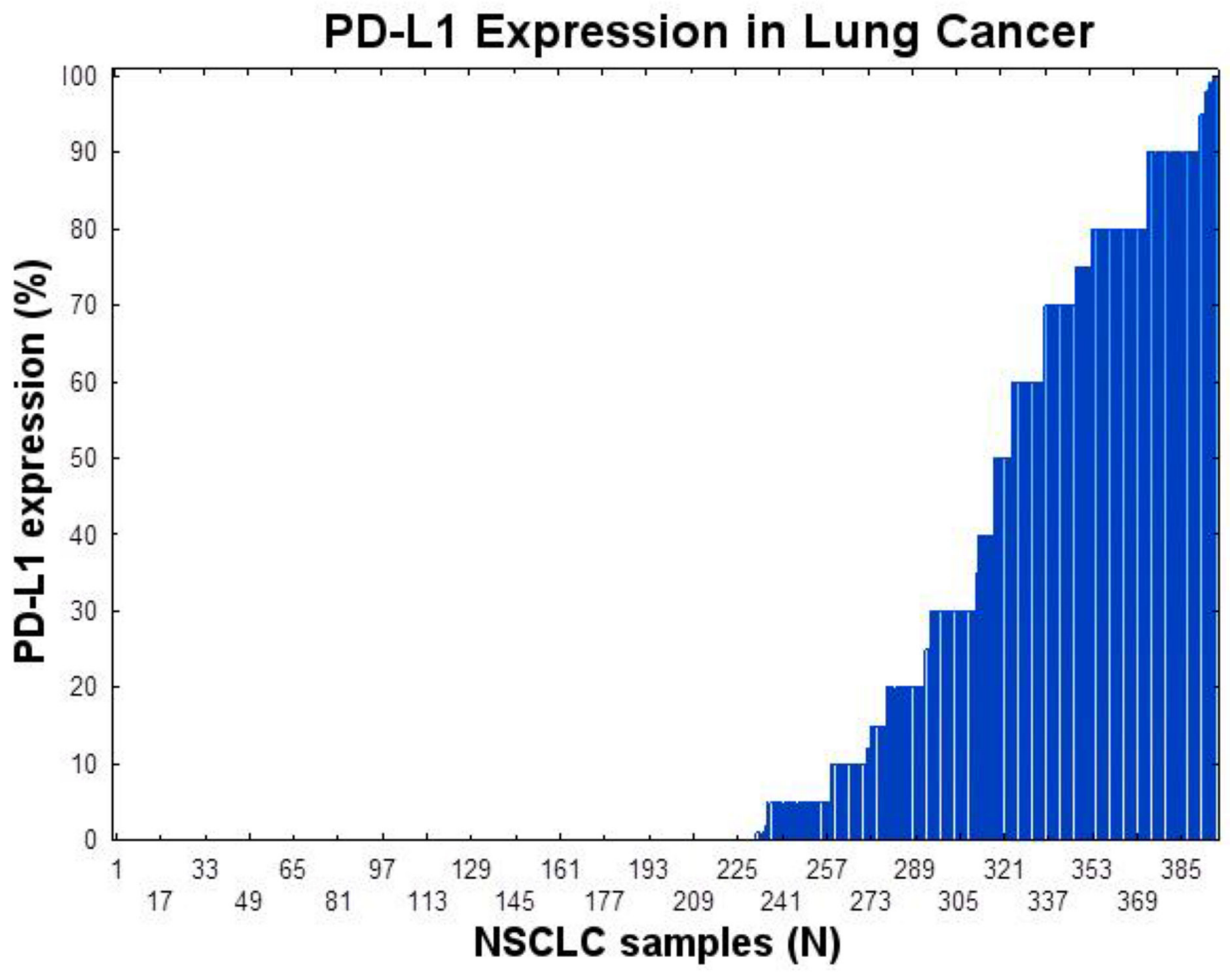
PD-L1 expression in NSCLC using immunohistochemistry using SP147 PD-L1 clone The tumor proportion score (TPS) is shown.

Positive PD-L1 expression (≥1%) appeared in 55.5% of males and 56.0% of females. Representative examples of PD-L1 protein expression by IHC are shown in Figure 1A-1D: A-C shows different staining of the same tumor with panel C representing PD-L1 staining and panel D shows intermediate PD-L1 staining of a different tumor. As shown in Figure 2, PD-L1 (SP 142) was expressed in 166/397 (41.8%) of NSCLC patients tested in this study. Twenty seven (7%) had expression between 1-5%, 61 (15%) between 1-20%, 92 (23%) between 1-50%, and 74 (18.6%) had PD-L1expression > 50% in tumor cells (TPS; tumor proportion score).

### Inverse correlation between *EGFR* mutation and PD-L1 expression

**Figure 3:**
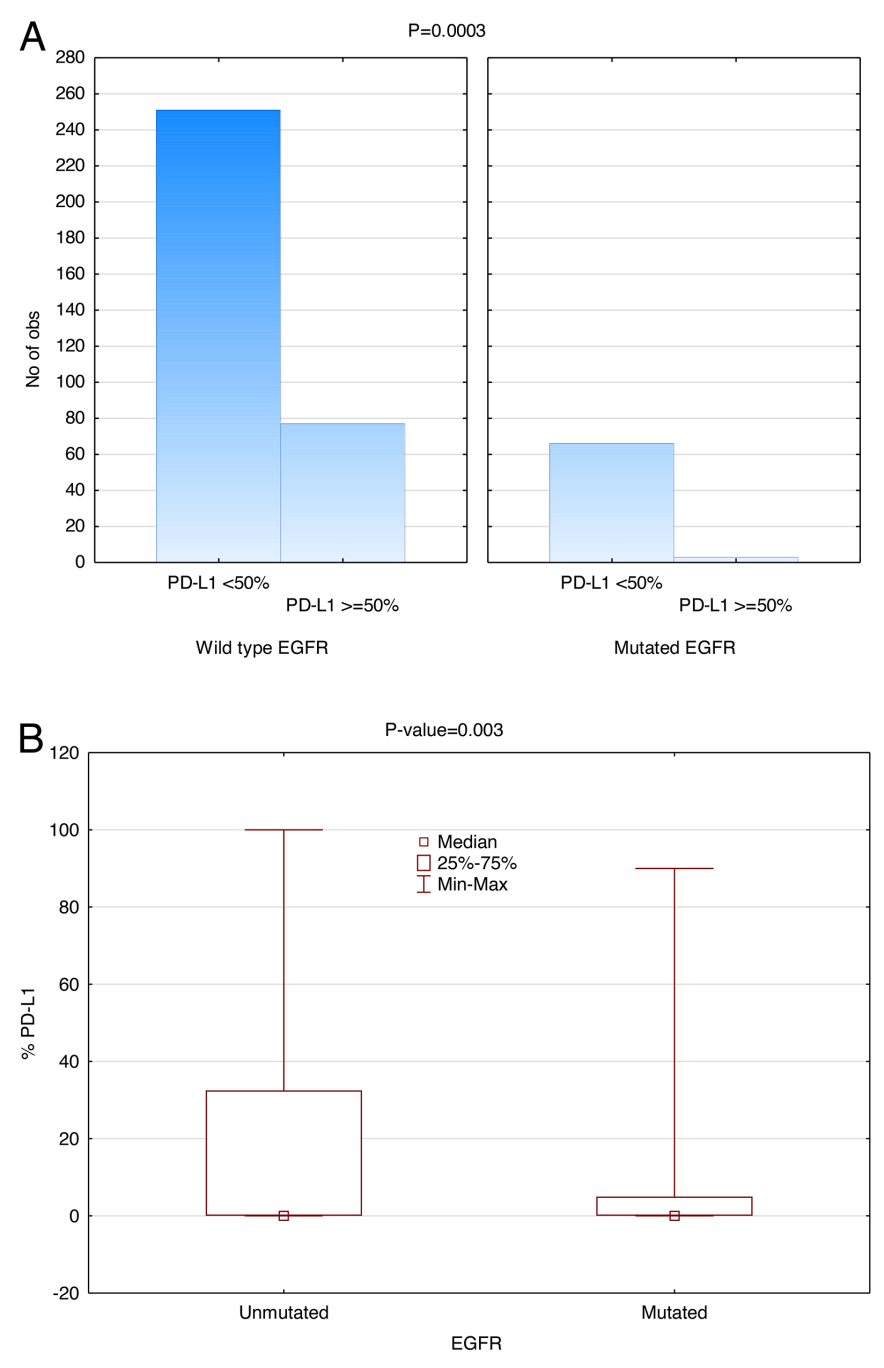
Inverse correlation between *EGFR* mutation and PD-L1 Expression (SP142) Significantly fewer patients with *EGFR* mutation had PD-L1 TPS>50% (P=0.0003, Wilcoxon rank-sum test). **(A)** The height of the histogram represents the number of patients. The two panels compare *EGFR* WT on the left with mutant *EGFR* on the right. Within each panel, the first data point represents the number of patients expressing PD-L1 less than 50% and the second data point represents the number of patients expressing PD-L1 more than or at 50%. **(B)** *EGFR* versus PD-L1 expression as a continuous variable in box plot form.

Next generation sequencing (NGS) revealed that 17.4% (69/397) patients had *EGFR* mutations in our study. Of the *EGFR* WT patients, 251 did not have PD-L1 expression (TPS <50%) and 77 (23.5%) did. Of the patients who did have *EGFR* mutations, 66 did not have PD-L1 expression (TPS <50%) and 3 did, representing 4.4% (3/69) PD-L1 expressing in *EGFR* mutated consort. As shown in Figure 3, the distribution of *EGFR* WT has higher PD-L1 expression than the mutated *EGFR*. The number of cases with *EGFR* mutation and PD-L1 expression ≥50% was small, but the data suggests that there is a reverse correlation between PD-L1 expression and *EGFR* mutational status even when considering PD-L1 expression as a continuous variable (P=0.0025). Additionally, when a cut-off point of 50% is used for PD-L1 expression, there were significantly fewer positive cases in the *EGFR* mutant as compared to *EGFR* WT (P=0.0003). This correlation becomes slightly weaker as the PD-L1 expression decreases (P=0.002 @ TPS=20% and P=0.137 @ TPS=5%).

### Positive correlation between *MET* amplification and PD-L1 expression

**Figure 4:**
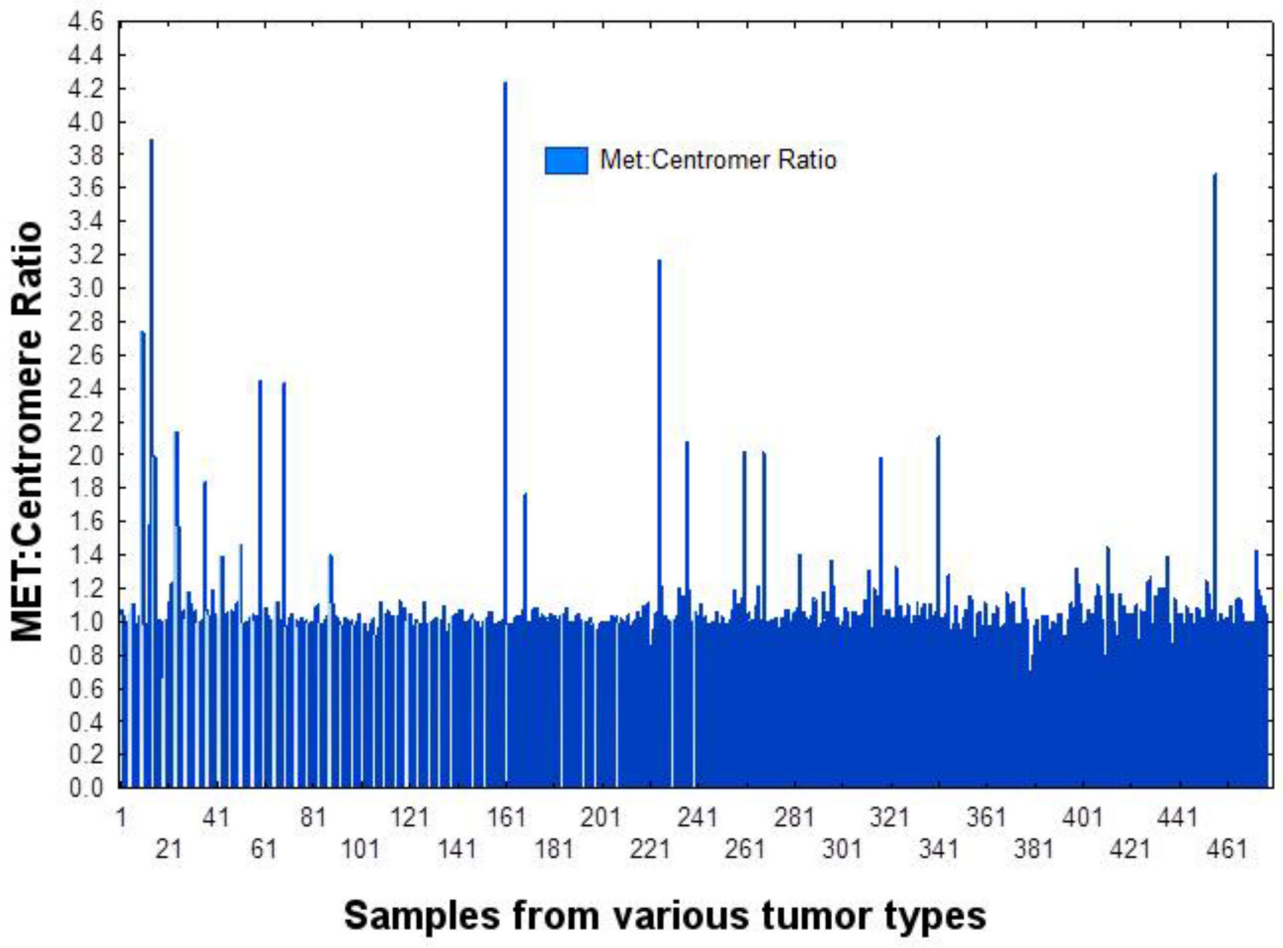
Establishing a cut-off point for *MET* Amplification by FISH (*MET*:centromere ratio of 1.5) Based on the signal to noise pattern show in this graph, a ratio ≥1.5 was determined to reflect amplification.

**Figure 5:**
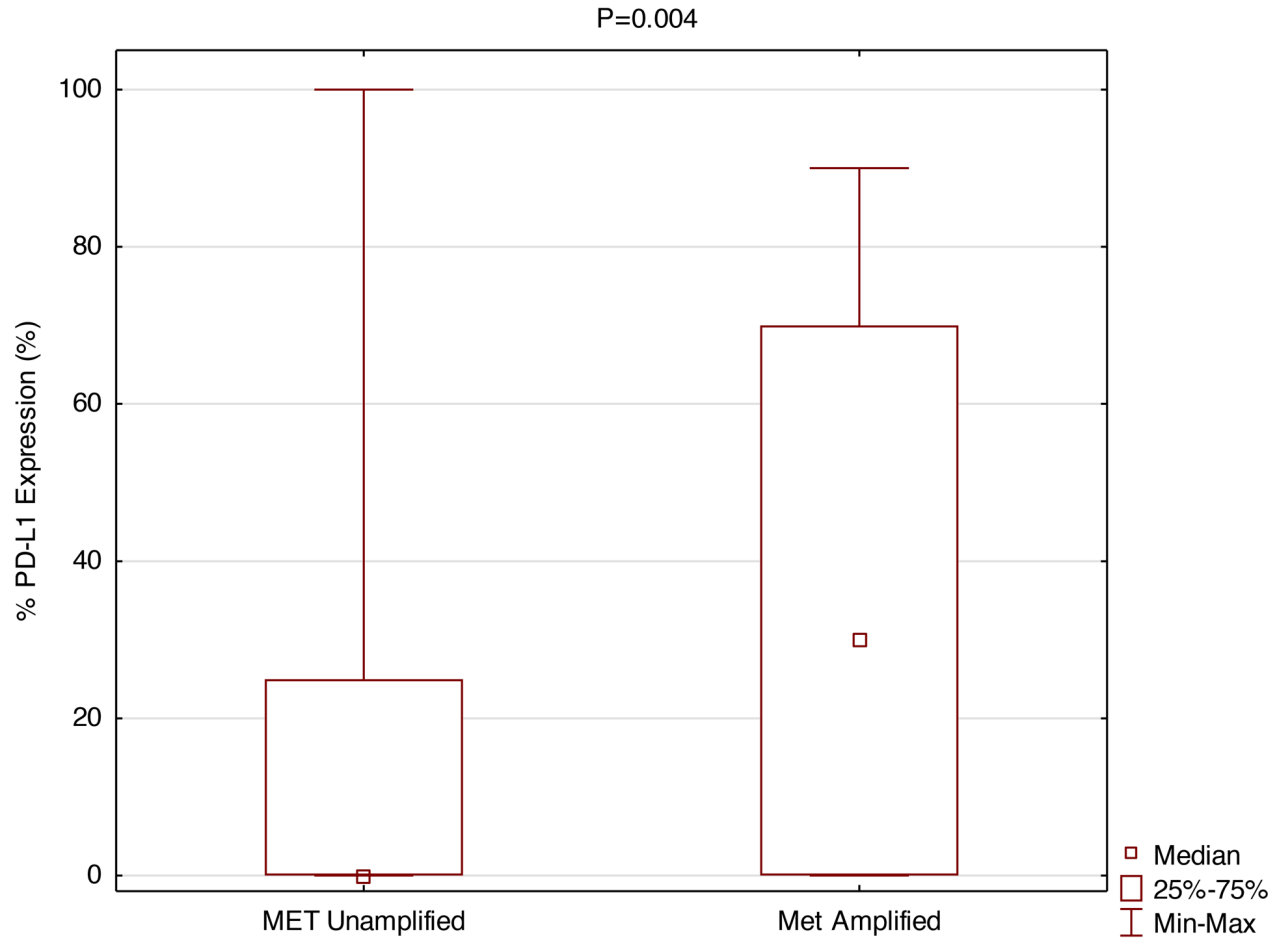
Positive correlation between *MET* amplification and PD-L1 Expression (SP 142) Patients with MET amplifications had significantly higher PD-L1 TPS (Wilcoxon rank-sum test). *MET* amplification versus PD-L1 expression as a continuous variable in box plot form.

As shown in Figure 1 and Figure 4, based on testing 471 patients with various tumors, including 397 NSCLCs, for *MET* copies by FISH as compared to chromosome 7 centromere, we established that a cut-off point (CO) of 1.5% is appropriate for determining *MET* amplification. Of the 397 patients measured for PD-L1 expression, 389 were also measured for *MET* amplification. *MET* was amplified (CO > 1.5%) in 4.1% patients with NSCLC. As shown in Figure 5, with *MET* ratio as the independent variable and PD-L1 as a continuous dependent variable, expression of PD-L1 is correlated with *MET* amplification. Those patients with *MET* amplification (≥ 1.5%) 10/16 (62.5%) had PD-L1 expression above the median, whereas for those patients without *MET* amplification (< 1.5%) 155/373 (41.6%) had PD-L1 expression above the median. Patients with *MET* amplification (CO > 1.5%) had a significantly higher (P=0.004) percentage of PD-L1 expression as a continuous variable as well as when cut-off points of 5% (P=0.01), 20% (P=0.0006), and 50% (P=0.01) are used.

### Positive correlation between *TP53* mutation and PD-L1 expression

**Figure 6:**
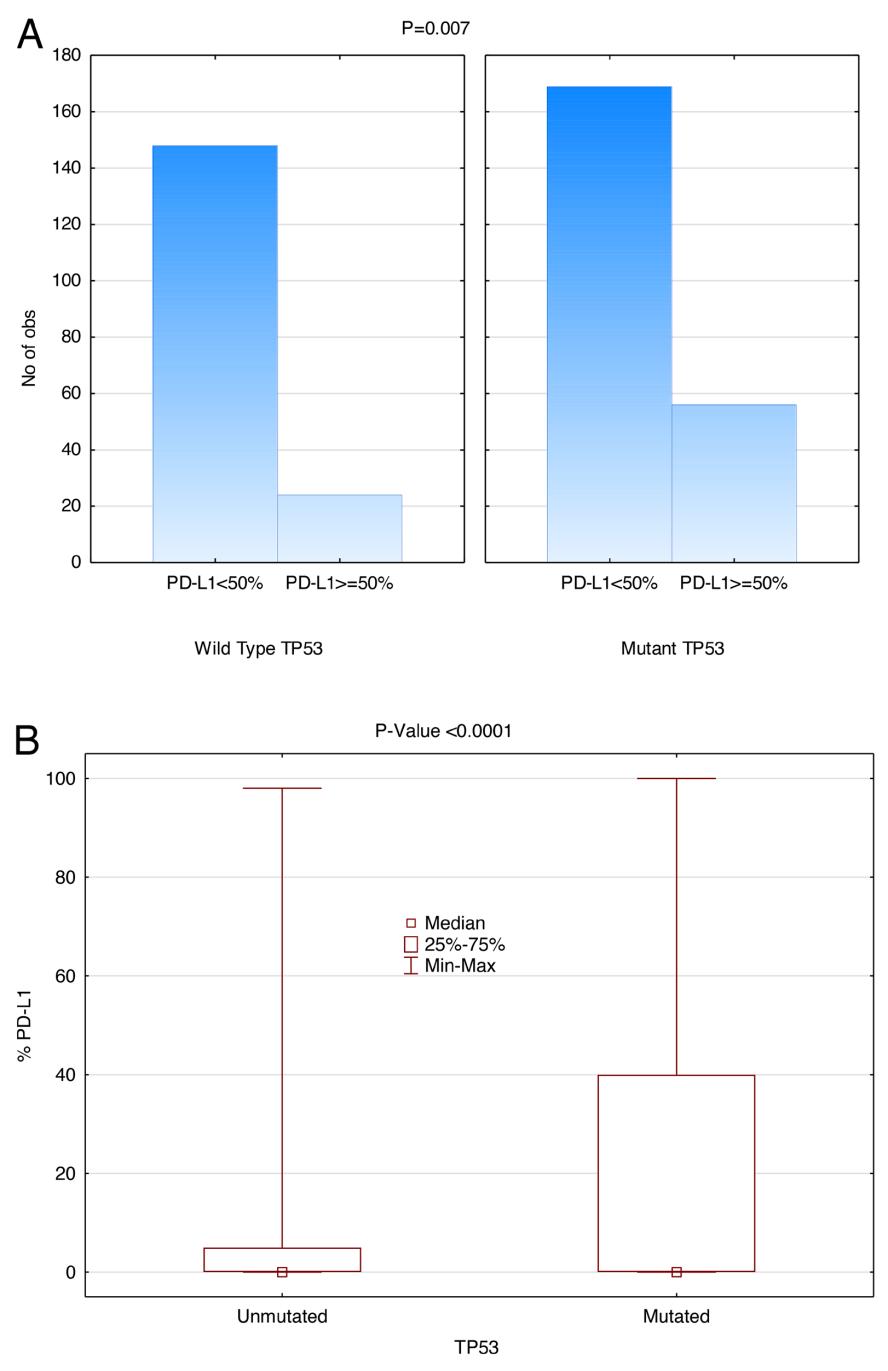
Positive correlation between *TP53* mutation and PD-L1 Expression (SP142) Patients with *TP53* mutation had significantly higher PD-L1 TPS (Wilcoxon rank-sum test). **(A)** The height of the histogram represents the number of patients. The two panels compare *TP53* WT on the left with mutant *TP53* on the right. Within each panel, the first data point represents the number of patients expressing PD-L1 less than 50% and the second data point represents the number of patients expressing PD-L1 more than or at 50%. **(B)** *TP53* versus PD-L1 expression as a continuous variable in box plot form.

NGS revealed that 225/397 (56.7%) of NSCLC patients had a *TP53* mutation. As shown in Figure 6, there was significant correlation between *TP53* mutation and overall PD-L1 expression (P<0.0001) when PD-L1 is used as a continuous variable. This positive correlation is also detected when PD-L1 expression cut-off points of 50%, 20%, or 5% are used (P=0.007, P=0.0003, and P=0.0004, respectively). For *TP53* WT, 24/172 (14.0%) were PD-L1 expressing (cut-off ≥ 50%) and 56/225 (24.9%) expressed PD-L1 for *TP53* mutated samples. Notably, *TP53* mutation was significantly more common in *EGFR* WT cases (P=0.0002).

### No correlation between *KRAS* mutation and PD-L1 expression

NGS revealed that 184/397 (46.3%) of NSCLC patients had *KRAS* mutations, including activating and nonactivating mutations. There was no correlation between *KRAS* mutation and overall PD-L1expression (P=0.49) when PD-L1 is considered as a continuous variable or for PD-L1 expression cut-offs 5%, 20%, or 50%. However, when *TP53* and *KRAS* were co-mutated, PD-L1 increased continuously (P=0.0002), and the co-mutation was significantly correlated with PD-L1 expression both at TPS of 5% (P=0.001) and 50% (P=0.0002).

## DISCUSSION

In this study we found that PD-L1 expressing *EGFR* WT NSCLC patients are statistically more likely to also overexpress *MET* than those who do not express PD-L1. Although measuring *MET* amplification in NSCLC is valuable for treatment decisions, it is difficult to quantify, with a limited number of putative *MET*-amplified patients. *MET* amplification occurs in approximately 2-4% of patients with lung adenocarcinoma, higher in other histologic types of lung cancer, and up to 22% of patients with acquired resistance to EGFR inhibition therapy [27–29]. We were able to establish that a cut-off point (CO) of 1.5% is appropriate for determining *MET* amplification, which compares favorably with earlier studies [29]. In our study, *MET* was amplified in 4.1% of NSCLC patients, and patients with *MET* amplification had significantly (P=0.004) more PD-L1 expression.

Our results indicate that *EGFR* mutation is inversely correlated with PD-L1 expression in NSCLC. *EGFR* WT patients included 23.5% that expressed high PD-L1, while *EGFR* mutated patients included only 4.4% that expressed high PD-L1. Some earlier studies also found this result: PD-L1 expression is higher in *EGFR* WT [30, 31]. Moreover, activation of the *EGFR* pathway has been shown to induce PD-L1 expression, [7, 32] while inhibition of EGFR has been shown to down-regulate expression of PD-L1 in *EGFR* mutated NSCLC cells, but not in the *EGFR* WT cells [12, 32]. This regulatory role of *EGFR* on PD-L1 expression, may explain the lack of PD-L1 expression by *EGFR*-mutant lung tumors and the lack of response rates reported for these patients [31]. Some previous studies reported the opposite findings: PD-L1 expression was higher in *EGFR*-mutant NSCLC [4, 11, 15, 33, 34]. Most of these studies included an *EGFR*-mutant patient cohort consisting predominately of patients who have received at least one cycle of EGFR tyrosine kinase inhibitor (TKI). This prior treatment may provide a rationale for the discrepancy, as a significant percentage of these patients may reasonably have acquired *MET* amplification after treatment, thereby driving PD-L1 expression secondarily. For *EGFR* WT patients, the relationship between *MET* amplification and PD-L1 expression may be critical for personalized cancer treatment that downregulates the *RAS/MAPK* pathway and where PD-L1 inhibitors are likely to be effective given the over-expression of PD-L1 seen in this patient population. A combination of MET targeting and checkpoint inhibitors may be particularly effective for *EGFR* WT patients, for whom other treatments are lacking.

The PD-1/PD-L1 pathway is a critical therapeutic target for advanced NSCLC, particularly in earlier disease settings. Although different anti-PD-L1 antibody clones are currently used in evaluating PD-L1 expression by immunohistochemistry (IHC), there is a concordance between antibodies for tumor cell scoring, but significant differences in the expression of PD-L1 and staining among 28-8, 22c3, SP142, and E1L3N, with SP142 producing the least stain [35, 36]. Various levels of PD-L1 expression have been noted in NSCLC studies depending on cut-off and clone [10]. Several investigators have suggested reporting both PD-L1 expression at any level in addition to expression ≥ 50% TPS by IHC staining [9]. However, the PD-L1 expression observed in this study using the SP142 clone was 41.8% (cut-off ≥ 1%) and 18.6% (cut-off ≥ 50%), and compares favorably with other studies that show similar results (e.g. 42.5% at any level and 18.4% with a PD-L1 expression level cut-off of ≥ 50%) [37, 38].

In our study, 46.7% of NSCLCs had *KRAS* mutations. In other studies, *KRAS* mutation was seen in 6-39%, varying with ethnicity, histology, and smoking status [39–41]. There was no correlation between *KRAS* mutation and overall PD-L1 expression (P=0.4). Also there was no correlation between *KRAS* mutation and PD-L1 when 5%, 20%, or 50% cut-off points were used. Other studies also failed to find a correlation between *KRAS* mutation and PD-L1 expression, [30, 34] though a few studies have found a significant positive correlation [11, 42]. Although it has not been clear why there have been discrepant results, concurrent dysregulation of downstream pathways, such as *TP53*, in contributing to the regulation of PD-L1 against the background of *KRAS* mutation, may help to explain the findings [42, 43]. In order to test this hypothesis, we calculated the expression of PD-L1 against combinations of *TP53* and *KRAS* mutant and WT. Indeed we found that although there was no correlation between *KRAS* mutant status and PD-L1 expression overall or in the presence of WT *TP53*, the co-mutation of *KRAS* with *TP53* was highly correlated with PD-L1 expression. Multiple studies have shown that mutations in *TP53* induce PD-L1 expression. This induction is reported be mediated by miR-34. On the other hand, PD-L1 expression has been reported to be suppressed by EGFR kinase inhibitors. Furthermore, MET overexpression is reported to be associated resistance to EGFR inhibitors. This may explain our findings.

*MET* amplification drives constitutive activation of the *RAF/MAPK* pathway, as do *KRAS* and *EGFR* mutations. Not surprisingly, except for two patients in our study, all samples with *KRAS* mutation were *EGFR* WT. *TP53* mutation, on the other hand, was correlated to both *EGFR* WT patient status and PD-L1 expression. Moreover, *TP53* is more common in *EGFR* WT cases, and together these data point to *TP53* being a driver of PD-L1 expression. In this study, *EGFR* mutant NSCLCs have significantly lower PD-L1 expression, but those without *EGFR* mutation have higher levels of PD-L1, *TP53* mutation, and MET activation; patients with *EGFR* WT NSCLC who overexpress PD-L1 are statistically more likely to also overexpress *MET* and have mutated *TP53*.

It has been shown previously that patients with *TP53* mutations are significantly more common in *EGFR* WT cases (P=0.0002) [44]. In our study, NGS revealed that 56.7% (225/397) of NSCLC patients had a *TP53* mutation, a bit higher than described in earlier reports (45-46%) [45]. Patients with *TP53* mutation had strikingly higher PD-L1 expression using the SP142 clone. In our study patients with *TP53* mutations had a higher copy number of the *MET* gene (CO ≥ 1.5%; P = 0.01), in keeping with other studies [44]. *TP53* mutations are seen more frequently in earlier stages of NSCLC [46, 47]. *TP53* has also been previously suggested to modulate the expression of immuno-regulatory genes, such as the TLR family and PD-L1 [48, 49]. Gain of function *TP53* mutation may play a role in metastasis early in the disease, suggesting patients harboring *TP53* mutation may benefit from personalized combination therapy. Because *TP53* mutations allow for the accumulation of a wide variety of mutated proteins, neoantigens, checkpoint inhibition and immune stimulation is an obvious choice.

Our data suggests that in NSCLC, both *MET* and *TP53* genes play a direct role in up-regulating PD-L1 expression. *TP5*3 mutation may play a role in metastasis early in the disease, suggesting patients without *EGFR*, *ALK*, or *ROS1* mutations, but harboring a *TP53* mutation, belong to a unique population subset that may benefit from combination therapy. Using PD-L1 or other immuno-oncology approaches in early stage NSCLCs, particularly those with *TP53* mutations, may delay metastasis, and allow conventional treatment to be more effective. Additionally, combination therapy targeting MET with checkpoint inhibition or TP53 with checkpoint inhibition should be considered in treating *EGFR* WT, *TP53* and/or *MET* amplified NSCLC.

## MATERIALS AND METHODS

### Patients, samples, and study approval

In this retrospective study, tissue samples were collected from 471 patients with various tumors, including 397 lung cancers. The median age of the females was 66 and 68 for males (P=0.04). There were 389 patients with available data on both *MET* amplification and PD-L1 expression. NSCLC patients in this retrospective study had *TP53* mutations (57%), *KRAS* mutations (46.7%), PD-L1 overexpression (41.8%), *EGFR* mutations (17.4%), and *MET* amplifications (4%). A summary of correlations uncovered in this study are summarized in Table 1. The present studies were reviewed and approved by the institutional review board and all were performed in accordance with relevant guidelines and regulations. The histological subtype of tumors was confirmed by a board-certified pathologist with expertise in thoracic malignancies.

**Table 1:** Correlations among NSCLC genomic markers and PD-L1. Wilcoxon rank-sum test was used for generating the P-values

	P-value	q-Value^*^	PD-L1(TPS^+^)
**EGFR/PD-L1**	0.0025	0.01	continuous
	0.0137	0.05	5%
	0.002	0.009	20%
	0.0003	0.001	50%
**MET/PD-L1**	0.004	0.01	continuous
	0.01	0.05	5%
	0.0006	0.001	20%
	0.01	0.05	50%
**TP53/PD-L1**	<0.0001	<0.0001	continuous
	0.0004	0.001	5%
	0.0003	0.001	20%
	0.007	0.01	50%
**KRAS+TP53/PD-L1**	0.0002	0.0008	continuous
	0.001	0.01	5%
	0.0002	0.001	50%
**EGFR/TP53**	0.0002	0.001	N/A
**KRAS/PD-L1**	No Correlation		

^*^P-value after adjusting for multiple testing.

^+^Tumor proportion score.

Formalin 7μm fixed paraffin embedded sections were examined by a US-certified pathologist for tumor content, and circled for each patient. One slide was used for FISH testing. Four to six consecutive sections were scrapped by a trained licensed clinical technologist for DNA extraction. Only samples with > 20% tumor were used for testing.

### PD1/PD-L1 IHC

PD-L1 expression on 397 lung cancer samples was evaluated with IHC using the SP142 clone and standard immunohistochemistry procedure. The tumor proportion score (TPS) was determined by calculating the percentage of tumor cells showing partial or complete membrane staining at any intensity. PD-L1 expression in tumor cells was considered positive if ≥1% of tumor cells had membranous staining of any intensity and high if ≥50%. Biopsies were reviewed and scored by trained/certified pathologists at NeoGenomics Laboratories. Membranous PD-L1 expression on tumor cells was defined by PD-L1 expression cut-offs of ≥50% (high) and 1%–49% (positive) [6].

### Next generation sequencing analysis

Lung cancer samples from 397 patients were also sequenced using next generation sequencing (NGS) for mutations in *TP53*, *KRAS*, and *EGFR*. DNA was sequenced using Illumina NGS protocols, including Illumina TruSeq library preparation, Illumina sample indexing, and Illumina synthesis by sequencing (SBS) protocols as recommended by the Illumina (San Diego, CA, USA). In brief, tumor DNA was amplified using either TruSeq kit or custom primers, and amplification products were confirmed with gel electrophoresis using a 2% agarose E-gel (Thermofisher, Carlsbad, CA, USA). Samples were indexed and pooled. Libraries were then loaded on to an Illumina MiSeq (Illumina, San Diego, CA, USA) or Nextseq Instrument for SBS using 150 bp Illumina sequencing kit with Illumina midoutput flow cells. An experiment sheet was generated using Illumina Experiment Manager for each sequencing run. MiSeq Reporter was used for alignment and variant calling using the proper panel bed/manifest file. Exons 2, 3, and 4 of *KRAS* were sequenced. For *EGFR*, we sequenced exons 3, 7, 15, and 18–21. Exons 1 and 3-7 of *TP53* were sequenced. The primers for targeted sequencing covered approximately 50 nucleotides from each side of each exon. Sequencing and library quality were assessed for every run using MiSeq reporter, which calculates amplicon read coverage per sample and uniformity of coverage. Positive and negative control samples were also sequenced in parallel with each run to confirm the sensitivity and specificity of each run. Overall sequencing quality was also assessed with MiSeq Reporter software. The average sequencing coverage across the entire coding regions was 10,000 in 94% of the sequenced amplicons. Only clinically significant and biologically relevant mutations were considered. Mutations of unknown significance were not considered.

### *MET* amplification

In this retrospective study, tissue sampled collected from 471 patients with various tumors, including 397 lung cancers, were tested for *MET* gene amplification by FISH by using a *MET* (7q31) probe and centromere 7 as a control (Agilent, Santa Clara, CA). Signals were quantified and ratios were calculated.

### Statistics

Standard statistical tests were used to evaluate correlations between variables including: Chi-square and Kruskal-Wallis test.
